# Influence of silicone oil tamponade on self-sealing sclerotomy using 25-gauge transconjunctival sutureless vitrectomy: a retrospective comparative study

**DOI:** 10.1186/s12886-015-0159-z

**Published:** 2015-12-01

**Authors:** Hirotsugu Takashina, Akira Watanabe, Hiroshi Tsuneoka

**Affiliations:** Department of Ophthalmology, National Hospital Organization Sagamihara Hospital, 18-1 Sakuradai, Minami-ku, Sagaminara, Kanagawa 252-0392 Japan; Department of Ophthalmology, Jikei University School of Medicine, 3-19-18 Nishi-shinbashi Minato-ku, Tokyo, 105-8471 Japan

**Keywords:** Self-sealing, Silicone oil injection, Silicone oil removal, Sclerotomy leakage, Hypotony

## Abstract

**Background:**

Characteristic complications have been reported for transconjunctival sutureless vitrectomy, such as postoperative sclerotomy leakage and postoperative hypotony. Particular attention to sclerotomy closure is required in cases of silicone oil tamponade, because postoperative supplementation of silicone oil implies reoperation, whereas postoperative supplement of gas is comparatively easy. This study investigated sclerotomy closure in cases of silicone oil tamponade using 25-gauge transconjunctival sutureless vitrectomy.

**Methods:**

We enrolled 19 consecutive eyes with silicone oil injection (Group A, self-sealing sclerotomies, *n* = 10) (Group B, sutured sclerotomies, *n* = 9) and 10 eyes with silicone oil removal (Group C, self-sealing sclerotomies) using 25-gauge TSV. Postoperative intraocular pressure was compared between Groups A and B, and between Groups A and C using repeated-measures analysis of variance (ANOVA), one-way factorial ANOVA, and the Tukey-Kramer test.

**Results:**

No significant differences in age or axial length were seen among groups, but surgical time differed significantly between Group C and the other groups. Mean duration of silicone oil tamponade was 3.2 ± 1.4 months in Group C, and no sclerotomies in Group A or C required suture placement. Postoperative silicone oil leakage to the subconjunctival space was not encountered in Group A. No cases showed postoperative hypotony (defined as intraocular pressure <5 mmHg). Significant differences in intraocular pressure within the same postoperative period were not identified between Groups A and B. Conversely, significant differences in intraocular pressure within the same postoperative period were identified at postoperative days 1 and 2, although not at postoperative week 1 or postoperative month 1 between Groups A and C.

**Conclusions:**

The procedure for sclerotomy closure seems to have little influence on postoperative intraocular pressure in eyes with silicone oil tamponade using 25-gauge transconjunctival sutureless vitrectomy, because silicone oil tamponade may avoid postoperative hypotony by decreasing sclerotomy leakage in the early postoperative period.

**Electronic supplementary material:**

The online version of this article (doi:10.1186/s12886-015-0159-z) contains supplementary material, which is available to authorized users.

## Background

The 25-gauge transconjunctival sutureless vitrectomy (TSV) first reported by Fujii et al. [1] in 2002 and the 23-gauge TSV described by Eckardt et al. [2] in 2005 have become more common in recent years. The characteristic procedure in TSV compared with conventional 20-gauge vitreotomy is sutureless sclerotomy; in other words, self-sealing sclerotomy. Potential advantages of TSV over conventional 20-gauge vitrectomy include faster wound-healing, reduced conjunctival scarring, greater patient comfort, decreased postoperative inflammation, reduced postoperative astigmatic changes, and shorter surgical opening and closing times [3]. However, characteristic complications of TSV have been reported to include postoperative sclerotomy leakage and postoperative hypotony [4–9], so surgeons have been apprehensive about the potential weakness of self-sealing sclerotomy. Preventing weakness of the self-sealing sclerotomy has been examined in several articles. Lin et al. [10] suggested that using an oblique sclerotomy incision rather than a conventional sclerotomy incision reduced the incidence of postoperative sclerotomy leakage because of its natural self-sealing effect, and Shimozono et al. [11] found that a three-step incision, similar to the self-sealing incision in cataract surgery, reduced postoperative hypotony. Amato et al. [4] suggested that leakage-related complications occurred, especially in previously vitrectomized eyes, due to the failure of peripheral vitreous to plug the unsutured sclerotomy. The mechanisms of self-sealing sclerotomy would thus appear to involve adherence between the internal and external valves and vitreous incarceration in the sclerotomy. Although the mechanisms underlying self-sealing sclerotomy appear to have been resolved, a fear of the weakness of self-sealing sclerotomy is still encountered. On the other hand, Cibis et al. [12] first reported silicone oil (SO) tamponade for vitreoretinal surgery in 1962, and SO tamponade has since seen wide use for severe vitreoretinal disease. Song et al. [13] described shrinkage of the vitreous basal gel in SO extraction cases because of previous tamponade. If much remnant vitreous remains, there will be a possibility of severe complications such as proliferative vitreoretinopathy after the surgery due to postoperative shrinkage of vitreous gel, so thorough intraoperative peripheral vitrectomy is required ahead of SO tamponade. However, performing thorough peripheral vitrectomy reduces vitreous incarceration into the sclerotomy, which is one of the self-sealing factors at the end of surgery. Colyer et al. [5] described a key concern with inferior rhegmatogenous retinal detachment as reduction in the efficacy of endotamponade due to postoperative leakage of gas through self-sealing sclerotomy. Similarly, postoperative leakage of SO through the sclerotomy may influence the success of surgery. Furthermore, we should pay particular attention to sclerotomy closure in cases of SO tamponade, because postoperative supplementation of SO implies reoperation, whereas postoperative supplement of gas is comparatively easy. This study examined sclerotomy closure in cases of SO tamponade using 25-gauge TSV.

## Methods

This retrospective study was approved by the institutional committee of National Hospital Organization Sagamihara Hospital, and complied with the tenets of the Declaration of Helsinki. Written informed consent was obtained from all patients after explanation of the study. We enrolled 19 consecutive eyes of 17 patients with injection of 1000 centistokes of SO (SILIKON1000®; Alcon Laboratories, Fort Worth, TX) and 10 eyes of 10 patients with removal of 1000 centistokes of SO (SILIKON1000®) treated using 25-gauge TSV at our hospital from May 2013 through August 2014. Patients who had undergone buckle procedure or gas tamponade were excluded. Each surgery was performed by the same surgeon (H.T.), and TSV was performed with the Alcon 25-gauge system (TOTAL PLUS®/Viscous Fluid Control Pak®/Constellation®; Alcon Laboratories). Oblique incisions were made vertical to the limbus at an angle of 45° to the sclera and cannulas were placed 3.5 mm posterior to the limbus using the 25-gauge EDGEPLUS® trocar/cannula system (Alcon Laboratories).

The sclerotomy for intraocular infusion (infusion site) was made in the inferotemporal quadrant, and sclerotomies for intraocular manipulation, such as using a pneumatic vitreous cutter, endolaser probe, and endoilluminator (manipulation sites), were made in the superonasal and superotemporal quadrants. Sclerotomies in vitrectomized eyes, such as SO removal, were placed in a position that sufficiently avoided the sclerotomy scar of the previous vitrectomy. Thorough peripheral vitrectomy and confirmation of remnant vitreous with a wide-angle viewing system (Resight®; Carl Zeiss, San Leandro, CA) and scleral indentation was performed to prevent complications caused by postoperative shrinkage of the remnant vitreous, and lensectomy was added in phakic eyes to allow thorough peripheral vitrectomy and to prevent SO-induced cataract formation [14]. At the end of surgery, scleral massage was performed to allow self-sealing of the sclerotomy in cases of SO injection from January 2014 through August 2014 (Group A, *n* = 10), while a suture was placed to close the sclerotomy in cases of SO injection from May 2013 through December 2013 (Group B, *n* = 9). Conversely, in all cases of SO removal, balanced salt solution (BSS Plus®; Alcon Laboratories) was exchanged directly, and scleral massage was performed to allow self-sealing of the sclerotomy (Group C, *n* = 10). Because all cases of SO removal were performed thorough vitrectomy at previous surgery, the condition of remnant vitreous in cases of SO removal were considered to more closely resemble the cases of SO injection than the cases in which thorough vitrectomy was not performed. The conditions of sclerotomy self-sealing were defined as no existence of increasing bleb formation and normal intraocular pressure (IOP) as checked by tactile examination. If these conditions were not met despite performing scleral massage for about 1 min, a suture was placed at the sclerotomy. Patients in whom SO was injected (Groups A and B) were asked to maintain a facedown position for a few days postoperatively, while patients who underwent SO removal (Group C) were not. The following two comparisons were performed in our study.

### Comparisons between Groups A and B

In these groups, SO was injected similarly, but the treatment of sclerotomy differed (Group A, self-sealing; Group B, suture placement).

### Comparisons between Groups A and C

In both these groups, treatment of sclerotomy involved self-sealing, but the material injected was different (Group A, SO; Group C, balanced salt solution).

Repeated-measures analysis of variance (ANOVA) was used to compare IOP at the following postoperative period in both groups: postoperative day (POD)1, POD2, postoperative week (POW)1, and postoperative month (POM)1. Furthermore, if a significant difference was identified, one-way factorial ANOVA and the Tukey-Kramer test were used to pinpoint which factors were significantly different. Statistical analysis was performed using statistical software programmed by Hisae Yanai (Statcal-3; OMS Publication, Saitama, Japan) along with Excel software (Microsoft, Redmond, WA). Values of p less than 0.05 were considered statistically significant.

## Results

**Fig. 1:**
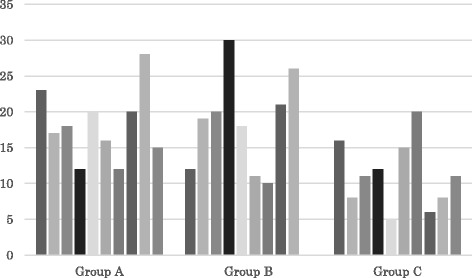
All cases of IOP at POD1 (mmHg). Intraocular pressure at postoperative day 1 differed significantly between Group C and the other groups (*p* < 0.05; Tukey-Kramer test)

The indications for surgery are shown in Table 1 and mean values for each factor are summarized in Table 2. Mean axial length was 24.7 mm (range, 22.62–32.64 mm) in Group A, 25.2 mm (range, 22.05–32.45 mm) in Group B, and 23.9 mm (range, 22.05–26.92 mm) in Group C. On the other hand, mean surgical time was 78.4 min (range, 44–124 min) in Group A, 70.9 min (range, 32–105 min) in Group B, and 36.2 min (range, 26–53 min) in Group C. No significant differences were seen among groups in age (*p* = 0.74; one-way factorial ANOVA) or axial length (*p* = 0.56; one-way factorial ANOVA), but surgical time differed significantly between Group C and the other groups (*p* < 0.05; Tukey-Kramer test). Mean duration of SO tamponade was 3.2 ± 1.4 months in Group C. No sclerotomies in Group A or C required suture placement. Postoperative SO leakage to the subconjunctival space was not encountered in Group A. In cases of combined surgery (phacoemulsification and vitrectomy), intra- or postoperative complications of anterior segment such as hyphema or papillary block due to fibrinous iritis were not occurred. Postoperative IOPs in each period are summarized in Table 3, and a column graph for all cases at POD1 are provided in Fig. 1, with no cases showing postoperative hypotony (defined as IOP <5 mmHg). In comparisons of IOP using repeated-measures ANOVA, no significant differences were identified between Groups A and B for either variation between subgroups (*p* = 0.80) or variation within subgroups (*p* = 0.72), while a significant difference was noted between Groups A and C for variation between subgroups (*p* < 0.05), but not for variation within subgroups (*p* = 0.24). Additional statistical examination using one-way factorial ANOVA and the Tukey-Kramer test for the significant difference (variation between subgroups between Groups A and C) proved that significant differences in IOP within the same postoperative period (*p* < 0.05) had existed at POD1 and POD2, although not at POW1 or POM1.Additional file 1 (age, surgical time, axial length,and postoperative IOP in each case) is available.

**Table 1:** Indications for surgery

	PVR	PDR
Group A SO injection/self-sealing	6	4
Group B SO injection/suture placement	7	2
Group C SO removal/self-sealing	5	5

**Table 2:** Mean and range for each factor

Group	Age (years)	Surgical time (min)	Axial length (mm)
A	57.4 ± 15.1 (39 ~ 77)	78.4 ± 23.3 (44 ~ 124)	24.7 ± 3.0 (22.62 ~ 32.64)
B	62.7 ± 19.8 (19 ~ 89)	70.9 ± 24.5 (32 ~ 105)	25.2 ± 3.1 (22.05 ~ 32.45)
C	61.7 ± 12.4 (39 ~ 77)	36.2 ± 8.2 (26 ~ 53)	23.9 ± 1.5 (22.05 ~ 26.92)

**Table 3:** Postoperative intraocular pressures in each period

Group	POD1 (mmHg)	POD2 (mmHg)	POW1 (mmHg)	POM1 (mmHg)
A	18.1 ± 4.9 (12 ~ 28)	17.4 ± 5.2 (12 ~ 27)	16.6 ± 5.6 (11 ~ 27)	17.4 ± 5.1 (12 ~ 29)
B	18.6 ± 6.8 (10 ~ 30)	16.4 ± 4.3 (8 ~ 23)	19.2 ± 4.6 (14 ~ 26)	17.1 ± 3.7 (11 ~ 24)
C	11.2 ± 4.7 (5 ~ 20)	10.4 ± 3.1 (6 ~ 16)	14.0 ± 4.0 (10 ~ 23)	14.4 ± 2.5 (11 ~ 18)

## Discussion

Severe sclerotomy leakage was not considered to be present postoperatively in this study, because no cases showed postoperative hypotony, and the sclerotomies in Groups A and C had achieved self-sealing with only restoration of the valve architecture because of the minimal vitreous incarceration due to thorough vitrectomy. However, Kapran et al. [15] reported a decrease in IOP caused by subclinical amounts of leakage in cases of self-sealing sclerotomies at 2 h postoperatively, although no significant difference was found between pre- and postoperative IOP on and after day 1. Hypotony on the day of surgery causes ciliochoroidal detachment [16], and once ciliochoroidal detachment has developed, several weeks is usually required for resolution [17, 18]. A temporary decrease in IOP due to subclinical amounts of leakage could thus have caused slight ciliochoroidal detachment in Groups A and C. If so, subclinical amounts of leakage might influence IOP during a certain postoperative period.

### Comparison between Groups A and B (self-sealing vs. suture placement)

We expected that postoperative IOP would be significantly lower in Group A than in Group B, because subclinical amounts of leakage would be present in Group A due to self-sealing sclerotomy, but barely existed in Group B due to suture placement. However, no significant difference was found, and postoperative closure of self-sealing sclerotomy was largely comparable to that of sutured sclerotomy in the early postoperative period in the case of SO tamponade. This suggested two possibilities. First, the subclinical amounts of leakage might be so small that no significant difference existed. Second, SO tamponade might erase the influence of subclinical amounts of leakage on postoperative IOP; that is, SO tamponade might avoid postoperative hypotony by decreasing sclerotomy leakage.

### Comparison between Groups A and C (SO tamponade vs. BSS® tamponade in self-sealing sclerotomy)

Although Amato et al. [4] reported the weakness of sclerotomy self-sealing in vitrectomized eyes, no significant correlation between history of vitrectomy and degree of difficulty of self-sealing was identified in our previous study [19]. Furthermore, subclinical amounts of leakage might be similarly present in both groups due to the use of self-sealing sclerotomy. We therefore did not expect to find any significant difference in postoperative IOP between these groups. However, significant differences were seen on POD1 and POD2. These differences suggest that intraocular tamponade materials might influence the early postoperative closure of the self-sealing sclerotomy and the possibility that subclinical amounts of leakage exist. On the other hand, the subclinical amount of leakage or ciliochoroidal detachment was considered to improve gradually, to the point where no significant difference existed at POW1 or POM1. From these two observations, subclinical amounts of leakage existed in the early postoperative period and were decreased by SO tamponade. Shimada et al. [20] reported that despite not suturing the 25-gauge scleral wounds, thorough peripheral vitrectomy followed by fluid-air/gas exchange reduced the incidence of postoperative low intraocular tension. Yamane et al. [17] suggested that surface tension might have held the detached ciliary body down in gas-filled eyes. Similarly, surface tension at the sclerotomy exists in cases of SO tamponade because of hydrophobic material, but not with balanced salt solution, and therefore may reduce the incidence of postoperative low IOP.

As a result, SO tamponade may avoid postoperative hypotony by decreasing sclerotomy leakage in the early postoperative period. In fact, although Tahiri et al. [21] described long duration of surgery as influencing the early postoperative decrease in IOP, the IOPs on POD1 and POD2 in Group C, for which surgical time had been significantly shorter than in Group A, were significantly lower than those in Group A. This might support the theory that SO tamponade decreases sclerotomy leakage in the early postoperative period. Some limitations to the present study need to be considered. First, the sample size was small, because SO was not used particularly frequently. Second, the evaluation of vitreous incarceration into the sclerotomy was not entirely accurate, because perfect vitreous shaving and objective judgment of vitreous incarceration were impossible. Of course, some investigations in recent years have used optical coherence tomography to evaluate sclerotomy closure [16, 17]. Although optical coherence tomography is very useful to project a picture of the real sclerotomy, the scope for objective evaluations such as quantification is considered limited. We therefore used IOP to evaluate sclerotomy closure in the early postoperative period in this study. Third, postoperative IOP was not considered to perfectly reflect postoperative sclerotomy closure, because severe surgical stress causes ciliary dysfunction. However, no findings plainly suggested ciliary dysfunction, such as aqueous flare, in any cases in this study. Evaluation of postoperative sclerotomy closure using IOP therefore did not influence the result in this study. Fourth, Group C had already undergone vitrectomy, unlike Groups A and B. However, previous vitrectomy was considered to have had little influence on the results in this study, because at least 2 months had passed since SO injection surgery.

## Conclusions

In conclusion, the procedure for sclerotomy closure (self-sealing or suture placement) seems to have little influence on postoperative IOP in SO tamponade eyes using 25-G TSV, because SO tamponade may avoid postoperative hypotony by decreasing sclerotomy leakage in the early postoperative period.

## Additional file

Additional file 1:
**Each factor and postoperative IOPs in each period of each case.** (XLS 30 kb)

## Abbreviations

TSV: transconjunctival sutureless vitrectomy
SO: silicone oil
IOP: intraocular pressure
POD: postoperative day
POW: postoperative week
POM: postoperative month
